# A Multi-Omics Approach Uncovers Divergent Mechanisms of Asthma in Normal Weight and Obese Children

**DOI:** 10.3390/metabo16050333

**Published:** 2026-05-15

**Authors:** Ilhame Diboun, Harshita Shailesh, Shana Jacob, Mohamed A. Elrayess, Stefan Worgall, Younes Mokrab, Ibrahim Janahi

**Affiliations:** 1 Medical and Population Genomics Lab, Sidra Medicine, Doha 26999, Qatar; idiboun@sidra.org (I.D.); ymokrab@sidra.org (Y.M.); 2 Department of Pediatric Medicine, Division of Pulmonology, Sidra Medicine, Doha 26999, Qatar; hshailesh@sidra.org; 3 Analytical Chemistry Core Facility, Sidra Medicine, Doha 26999, Qatar; shana.john@uq.edu.qu; 4 Biomedical Research Centre, QU Health, Qatar University, Doha 2713, Qatar; m.elrayess@qu.edu.qa; 5 Department of Pediatrics, Weill Cornel Medical College, New York, NY 10021, USA; stw2006@med.cornell.edu; 6 Department of Genetic Medicine, Weill Cornell Medicine, Doha 24144, Qatar; 7 Department of Biomedical Science, College of Health Sciences, Qatar University, Doha 2713, Qatar; 8 Department of Pediatrics, Weill Cornel Medicine, Doha 24144, Qatar

**Keywords:** asthma, lipidomics, metabolomics, obesity, pediatrics

## Abstract

**Highlights:**

**What are the main findings?**

- Pediatric obesity-related asthma is associated with distinct metabolomic and lipidomic signatures as compared to normal weight asthma.
- These signatures correlate with different metabolic, physiologic, and immune pathways that affect disease pathogenesis.

**What are the implications of the main findings?**

- Metabolomics and lipidomic biomarkers can be of potential help in improving stratification, diagnosis, and personalized management of pediatric asthma.
- Identification of metabolic pathways provides novel targets for therapeutic intervention and biomarker development.

**Abstract:**

**Background**: Children with obesity-related asthma exhibit poorer symptom control and more frequent exacerbations than their normal-weight peers, but the underlying metabolic mechanisms are unclear. This study aimed to identify drivers of obesity-related asthma through untargeted plasma metabolomic and lipidomic profiling. **Methods**: Plasma was obtained from normal weight (NW) asthmatic (*n* = 95) and non-asthmatic (*n* = 67) and overweight/obese (OO) asthmatic (*n* = 99) and non-asthmatic (*n* = 100) children (6–17 years). We assessed metabolic and lipidomic differences between asthmatics and controls within each BMI group using orthogonal partial least squares discriminant analysis (OPLS-DA), examined overlap with the adult Qatar Biobank cohort, and mapped metabolic–clinical interactions using Gaussian Graphical Models. **Results**: In the fitted OPLS-DA models, separation between asthmatic and control groups was stronger in the NW group (R^2^Y = 0.72/0.52) than in OO (R^2^Y = 0.65/0.63) children. Asthma was associated with altered tricarboxylic acid (TCA) intermediates, ether-linked phosphatidylethanolamines, and sphingomyelins (SM) in NW, and with phosphatidylcholines, lysophosphatidylcholines, and phosphatidylethanolamines in OO. Integrating metabolomic, lipidomic, and clinical data revealed connections between altered SMs and interleukins, and TCA intermediates and electrolytes, all associated with elevated leptin in NW. An increased residual volume to total lung capacity ratio in OO was associated with phospholipid shifts. The overall dynamics in lipid metabolism with asthma, conditioned on BMI, was also observed in the adult Qatar Biobank cohort. **Conclusions**: Among NW children with asthma, we found enhanced TCA cycle activity and inflammation linked to altered SM metabolism, whereas in OO, the findings suggest oxidative stress arising from chronic obesity-related inflammation. These data reveal BMI-specific metabolic mechanisms of pediatric asthma that might inform precision approaches to disease management.

## 1. Introduction

Asthma is a chronic inflammatory lung disease that causes airway narrowing and breathing difficulties. It is the most common chronic lung condition in children, often developing before the age of 5 years [1]. Although its exact cause remains unclear, asthma likely arises from a complex interplay between genetic and environmental factors [2]. Obesity is increasingly recognized as a common comorbidity in children with asthma [3], defining a distinct obesity-related asthma phenotype marked by poorer symptom control and increased disease severity. Childhood obesity is defined as a body mass index (BMI) ≥ 95th percentile for children of the same age and sex. A recent systematic review and meta-analysis of data from over five million participants reported that, globally, about 15% of children and adolescents are overweight and more than 20% have excess body weight [4]. In the Middle East and North Africa region, pooled estimates suggest some of the highest levels worldwide, with combined childhood overweight and obesity prevalences reaching up to 49.4% in recent studies [5]. Beyond its prevalence, high BMI in children and adolescents is increasingly recognized as a leading contributor to death and disability, highlighting long-term cardiometabolic and respiratory consequences that extend into adulthood [6]. The relationship between obesity and asthma is of particular concern in the pediatric population. Numerous studies have demonstrated a positive association between higher BMI and an increased risk of asthma development and exacerbation in children [7,8]. The mechanisms underlying this association are not fully understood but might involve systemic inflammation, metabolic dysregulation, mechanical factors such as reduced lung volumes, dietary influences, and microbial dysbiosis [9,10].

Indeed, several metabolic pathways have been proposed to link obesity and asthma. Altered carbohydrate metabolism, including insulin resistance and hyperglycemia, can promote airway remodeling and epithelial damage in obesity-related asthma [10,11]. Obesity also increases oxidative stress and reduces nitric oxide bioavailability, an endogenous bronchodilator, potentially by lowering the L-arginine-to-asymmetric dimethylarginine ratio [12]. Metabolomics studies have revealed distinct metabolic signatures in obesity-related asthma compared with obesity or asthma alone, suggesting unique underlying mechanisms [13]. For example, in obesity-related asthma, analysis of exhaled breath condensate metabolomics revealed significant associations with alterations in methane, glyoxylate, dicarboxylate, and pyruvate metabolic pathways when compared to the overweight/obese without asthma and normal-weight asthma groups [14]. Pro-inflammatory cytokines such as IL-6, commonly elevated in obesity, may contribute to asthma severity and impaired lung function [15]. Moreover, metabolic disturbances associated with metabolic syndrome appear to exacerbate asthma independently of obesity [11]. Genetic factors also play a role: for instance, variants at the 17q21 locus reduce de novo sphingolipid synthesis [16], which has been linked to increased airway hyperreactivity [17]. Dysregulated sphingolipid metabolism is further reported in obesity, with elevated plasma or serum levels of ceramides, dihydroceramides, and sphingomyelins [18].

Despite these insights, current understanding of metabolic mechanisms in pediatric obesity-related asthma remains fragmented, limiting the ability to identify biomarkers or tailor treatments to this distinct asthma endotype. This necessitates a systematic exploration of metabolic signatures underlying the interaction between obesity and asthma in children. Given the dynamic nature of metabolomic data due to temporal variability, biological variability, and susceptibility to confounding, careful control of confounders, such as medication use, comorbidities, and cohort selection, is essential. A rigorously designed metabolome-wide association study (MWAS) in a well-characterized pediatric cohort is still lacking and is needed to identify metabolic alterations specific to asthma in obese children, thereby revealing potential therapeutic targets for this high-risk group.

To fill this knowledge gap, we compared plasma metabolomic and lipidomic profiles in 361 children stratified by asthma status and BMI. We assessed asthma-related molecular pathways in normal-weight and obese groups, integrating hormonal, interleukin, and lung function parameters measured within the same cohort. We further compared these pediatric findings with adult data from the Qatar Biobank (QBB) cohort to evaluate overlap in BMI-specific asthma mechanisms.

## 2. Materials and Methods

### 2.1. Study Approval

**Figure 1:**
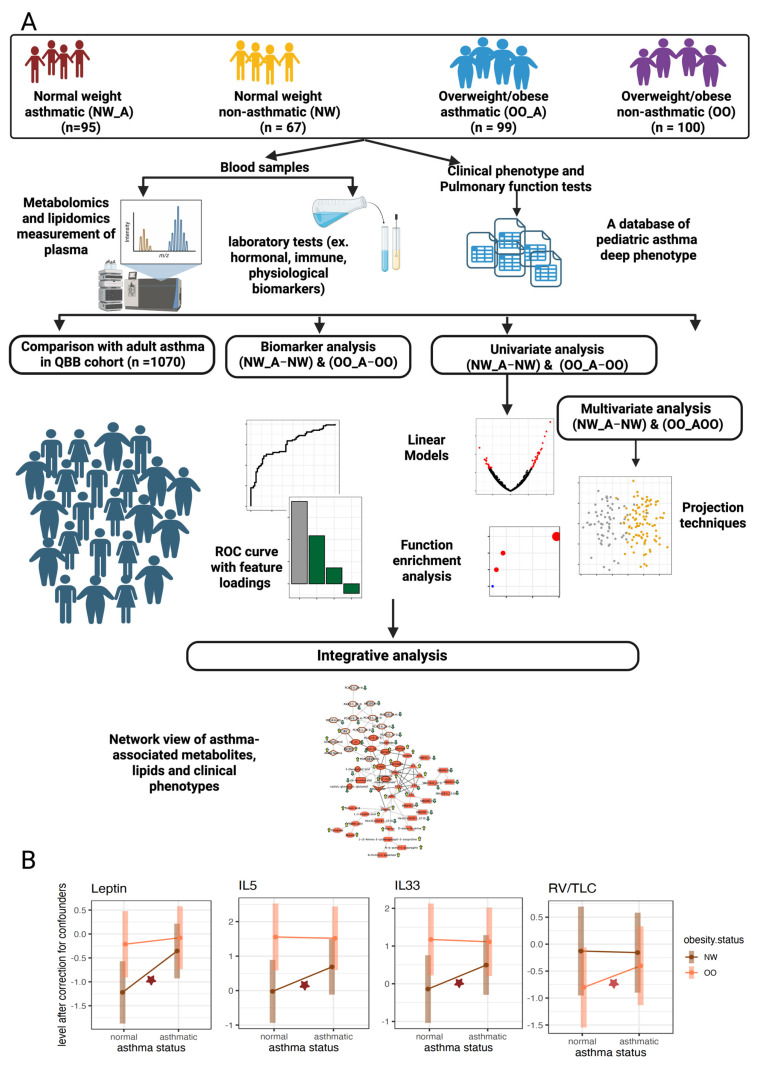
(**A**) Overview of the study design and analysis workflow. The cohort included four groups of children stratified by asthma status and BMI: normal weight asthmatic (NW_A), overweight/obese asthmatic (OO_A), normal weight non-asthmatic (NW), and overweight/obese non-asthmatic (OO). Plasma samples were analyzed using untargeted metabolomics/lipidomics and integrated with detailed clinical and pulmonary function data. (**B**) Selected clinical parameters significantly associated with asthma within specific BMI groups. Leptin, IL-5, and IL-33 levels were selectively elevated in NW_A, whereas the residual volume to total lung capacity ratio (RV/TLC) increased with asthma only in OO. The red star indicates nominal significance at *p* value ≤ 0.05.

This study forms part of the Sphingolipids in Obesity and Asthma in Pediatrics (SOAP) project, a large cross-sectional multi-omics study designed to investigate physiological, genetic, epigenetic, metabolomic, and lipidomic factors underlying obesity and asthma in children in Qatar. The study was approved by the institutional review board of Sidra Medicine (IRB No. 1500770). Detailed methods of the parent study have been previously described [19]. The design of the current study is summarized in (Figure 1A).

### 2.2. Patient Groups

Children aged 6–17 years were recruited between August 2017 and December 2022 from general pediatrics, pediatric pulmonology, and pediatric endocrinology clinics at Sidra Medicine, Doha, Qatar. A total of 361 participants were enrolled. Ethnicity was inferred from parent-reported nationality. Participants were classified into four groups according to BMI and asthma status: normal weight with asthma (NW_A, *n* = 95), overweight/obese with asthma (OO_A, *n* = 99), overweight/obese without asthma (OO, *n* = 100), and normal weight without asthma (NW, *n* = 67) (Figure 1A). An asthma diagnosis was made by the treating physician and confirmed only for subjects who had received the diagnosis at least 6 months before recruitment. Asthma was confirmed by a documented history of asthma symptoms according to the Global Initiative for Asthma (GINA) guidelines [20], regardless of previous lung function test results. Overweight and obesity were defined as a BMI at or above the 85th and 95th percentiles, respectively, for age and sex, based on World Health Organization criteria. Study exclusion criteria were as previously described [19]. Verbal or written assent was obtained from all participants, along with written consent from parents or legal guardians. Each participant underwent pulmonary function testing, and blood samples were collected to obtain plasma for metabolomics and lipidomics analyses.

### 2.3. Clinical Phenotype Measurements

Pulmonary function tests (spirometry and body plethysmography) were performed to measure forced vital capacity (FVC), forced expiratory volume in 1 s (FEV1), the FEV1/FVC ratio, forced expiratory flow at 25% and 75% of pulmonary volume (FEF 25–75%), peak expiratory flow (PEF), peak inspiratory flow (PIF) and forced expiratory time (FET). Lung volumes and resistance indices included specific airway resistance (sRAW), vital capacity (VC), inspiratory capacity (IC), functional residual capacity (FRCpleth), expiratory reserve volume (ERV), residual volume (RV), total lung capacity (TLC), and the RV/TLC ratio, as previously described [19]. Fractional exhaled nitric oxide (FeNO) and lung clearance index (LCI) were also measured for all participants following the same protocol [19]. Clinical parameters included plasma fasting glucose, 120-min postprandial glucose, serum lipids, fasting insulin, complete blood counts, standard biochemistry, vitamin D, and thyroid-stimulating hormone (TSH) for all subjects. In addition, HbA1c and C-peptide levels were assessed in OO and OO_A children [19].

### 2.4. Metabolomics and Lipidomics

20 µL of frozen plasma samples were thawed on ice in four batches of up to 94 samples, mixed, and centrifuged at 200× *g* for 1 min at 10 °C. Each sample was then aliquoted into two microcentrifuge tubes (30108051, Eppendorf, Hamburg, Germany): 75 µL for metabolomics analysis and 50 µL for lipidomics analysis. Additional aliquots from all samples were pooled in equal proportions to generate reference samples used for metabolite and lipid identification. An external plasma sample was included as a quality control (QC) sample.

### 2.5. Metabolomics Analysis

For metabolomics analysis, 15 µL of isotopically labelled QC standard mix (MSK-QC-KIT, Metabolomics QC Kit, Cambridge Isotope Laboratories, Tewksbury, MA, USA) was added to each plasma aliquot, followed by 450 µL of cold methanol (A456-212, Fisher Chemical, MA, USA). QC samples were prepared identically, except without the addition of the labelled QC standard mix. Tubes were capped and stored at −20 °C for 2.5 h, then mixed and centrifuged for 5 min at 19,800× *g* and 10 °C. The supernatant was transferred to glass vials (186007201C, Waters, Milford, MA, USA) and evaporated to dryness in a vacuum centrifuge (Genevac EZ-2 Plus, Genevac Ltd., Ipswich, UK). Residues were stored at −80 °C until analysis.

Before analysis, samples were reconstituted in 45 µL of 1% methanol in water (W6500, Fisher Chemical), and 2 µL of each was injected into an ultra-high-pressure liquid chromatograph coupled to an Orbitrap Tribrid mass spectrometer (Vanquish LC and Fusion Lumos, Thermo Scientific, Waltham, MA, USA). The chromatograph operated with two binary pumps running in parallel to condition the columns between injections. Two Accucore C18+ reverse-phase columns (27101–152130, 2.1 × 150 mm, 1.5 µm, Thermo Scientific, Waltham, MA, USA) were maintained at 45 °C using a mobile phase gradient of 0.1% aqueous formic acid (A1171, Fisher Chemical) and methanol with 0.1% formic acid (mobile phases A and B, respectively) at a flow rate of 0.2 mL/min. The mobile phase began at 1% B (0.5 min), increased to 60% B (5.5 min), then to 98% B (1.5 min), held for 4.6 min, raised to 100% B (0.1 min), and held for 1.3 min. During column conditioning, the columns were washed with isopropanol–acetonitrile (9:1, *v*/*v*; A461-212, A955-212, Fisher Chemical) for 5 min before returning to initial conditions. All positive electrospray ionization (ESI) injections were performed on one column and negative ESI injections on the other.

The heated electrospray ion source (NGS-HESI) was operated with sheath gas flow of 40 AU, auxiliary gas flow of 8 AU, sweep gas flow of 1 AU, and spray voltage of 3.5 kV (positive) or 3.0 kV (negative). The vaporizer and ion transfer tube were maintained at 320 °C and 275 °C, respectively. The automatic gain control (AGC) target was set to 1 × 10^5^ ions, with a 50 ms maximum injection time and an RF lens setting of 30%. Each sample was injected twice sequentially in positive and negative ESI modes across a mass range of +ESI 59–800 *m*/*z* and −ESI 70–800 *m*/*z*. All samples and QCs were acquired in full-scan mode with an Orbitrap mass resolution of 240K (FWHM at *m*/*z* 200), using internal calibration with fluoranthene ion.

QC samples were injected intermittently throughout the analytical run, and pooled samples were analyzed multiple times using AcquireX deep-scan mode to maximize MS2 fragmentation. Fragmentation was performed using higher-energy collisional dissociation (HCD) for peaks above an intensity threshold of 2E4. Data-dependent MS/MS acquisition used Orbitrap resolutions of 60K (MS1) and 30K (MS2), with stepped collision energies of 20, 35, and 50%, a maximum injection time of 100 ms, and an AGC target of 1.25 × 10^2^. Dynamic exclusion was enabled, with isotope and feature exclusion for 2.5 s after a single MS2 scan and a mass tolerance of 2 ppm.

### 2.6. Lipidomics Analysis

For lipidomics analysis, 20 µL of isotopically labelled lipid QC mix (330707, Splash Lipidomix, Avanti Polar Lipids, Alabaster, AL, USA) and 230 µL of cold isopropanol (A461-212, Fisher Chemical) were added to each plasma aliquot. The mixture was gently mixed and incubated at −20 °C for 1 h. Samples were then centrifuged for 5 min at 19,800× *g* and 10 °C, and the supernatant was transferred to glass vials, evaporated to dryness in a vacuum centrifuge, and stored at −80 °C until analysis.

On the day of analysis, samples were reconstituted in 90 µL of isopropanol–methanol:chloroform (1:1:1, 650498, Sigma Aldrich, Dermstadt, Germany). Each sample (1 µL injection volume) was injected as described above, using a column temperature of 70 °C. The mobile phases consisted of acetonitrile–water (6:4, *v*/*v*; MPA) and acetonitrile–isopropanol (1:9, *v*/*v*; MPB), each containing 0.1% formic acid and 10 mM ammonium acetate, with a flow rate of 0.25 mL/min. The gradient began at 10% MPB, held for 0.2 min, increased to 50% over 1.8 min, then to 100% over 23 min, and was maintained for 1.2 min. For negative ESI injections, chromatography was run under the same gradient up to 15 min. After each injection, columns were equilibrated to the initial mobile phase conditions using an auxiliary pump.

The HESI source was operated with sheath gas flow of 40 AU, auxiliary gas flow of 7 AU, sweep gas flow of 1 AU, and spray voltages of 3.5 kV (positive mode) and 2.8 kV (negative mode). The vaporizer and ion transfer tube were set to 320 °C and 350 °C, respectively. The automatic gain control (AGC) target was 1 × 10^5^ ions, with a maximum injection time of 25 s and an RF lens setting of 45%. Each sample was injected twice in ESI positive and negative modes, acquired at an Orbitrap mass resolution of 240K, over a full scan mass range of 200–1800 *m*/*z*.

The pooled QC sample was injected multiple times in AcquireX deep-scan mode to maximize MS2 fragmentation. Fragmentation was performed in HCD mode at an Orbitrap mass resolution of 120K (MS1) and 30K (MS2), using stepped collision energies of 25, 35, and 50%. Additionally, collision-induced dissociation (CID) at 35% collision energy was triggered upon detection of the phosphocholine headgroup (*m*/*z* 184.0733) and neutral loss fragments of M + NH_4_ adducts of common fatty acids.

### 2.7. Data Processing

Raw data files were processed using Compound Discoverer (version 3.3, Thermo Scientific, Waltham, MA, USA). Features were aligned and grouped with a mass tolerance of 3 ppm for metabolomics and 5 ppm for lipidomics datasets, allowing a maximum retention time shift of 1 min.

For metabolomics data, compounds were detected using ion forms [M+H]+, [M+H−H_2_O]+, [M+K]+, and [M+Na]+ in positive mode, and [M−H]− and [M+HCOO]− in negative mode. Lipidomics data were annotated with LipidSearch (version 4.23, Thermo Scientific, Waltham, MA, USA) using ion forms [M+H]+, [M+NH_4_]+, [M+Na]+, [M+H−H_2_O]+, [M−H]−, [M+HCOO]−, and [M+CH_3_COO]−, with a parent mass tolerance of 5 ppm and fragment mass tolerance of 10 ppm.

Metabolite features were further annotated using multiple databases and matching criteria. An in-house MS^2^ library of over 950 compounds was used, matching elemental composition, retention time, and MS^2^ spectra. The mzCloud database was also used with matching based on elemental composition and MS^2^ spectra, both with parent mass tolerance of 5 ppm and fragment mass tolerance of 10 ppm. In addition, public databases including BioCyc, ChEBI, HMDB, KEGG, LipidMAPS and SMPDB were queried using the mzLogic algorithm with a mass tolerance of 3 ppm.

During processing, the Fill Gaps node (mass tolerance 3 ppm) was applied, and SERRF QC correction was implemented to normalize sample responses between batches, maintaining a maximum corrected QC area RSD of 20% within and across batches. All identified compounds were integrated for peak area in each sample and included in subsequent statistical analyses. Isotopically labelled internal standards were monitored for retention time stability, peak intensity, and mass accuracy. All monitored peaks remained within acceptable limits (±2% of median retention time, ±30% of median peak abundance, and ±3 ppm mass accuracy).

Additional metabolite and lipid annotations, including class affiliations and identifier mappings, were performed using MBROLE (version 2.0) [21], MetaCyc (version 29.6) [22], and R package RefMet (version 1.0.0) [23].

### 2.8. Statistical Analysis

#### 2.8.1. Sample Size Calculation

The sample size was determined based on pilot data assessing the serum ceramide-to-dihydroceramide ratio in children. A total of 90 subjects per group were required to detect a mean difference of 0.25 (unitless ratio) between non-atopic asthmatic children (mean 1.47) and non-atopic, non-asthmatic children (mean 1.22), assuming a standard deviation of 0.6, 80% statistical power, and a 5% significance level.

#### 2.8.2. Multivariate and Univariate Analysis

Metabolomics, lipidomics, and clinical data were normalized using the rank-based inverse normal transformation method [24]. Orthogonal partial least squares discriminant analysis (OPLS-DA) was performed using SIMCA software (version 18.0, Sartorius). This was performed on the metabolomics and lipidomics datasets separately for the OO and NW groups to assess separation between asthmatic and non-asthmatic subjects [25]. The R^2^Y and Q^2^ metrics were calculated to represent the total variance in phenotype explained and the model’s predictive performance, respectively, using cross-validation.

Univariate analysis was conducted using linear models in R statistical software (version 4.1.2) for both metabolomics and lipidomics data. Each model included an interaction term (obesity × asthma) and accounted for potential confounders including age and six medication classes (β_2_-adrenergic receptor agonists, corticosteroids, glucocorticoid, histamine antagonists, leukotriene receptor antagonists, and non-selective COX inhibitors). Additionally, BMI (expressed as percentile for age and sex) and sex were adjusted for within obesity groups. An additional confounder term was based on the orthogonal component identified through OPLS-DA of each dataset. The model used is outlined below:

Analyte~age + orthogonal_comp + medication_class1 + … + medication_class6 + obesity × sex + obesity × bmi + obesity × asthma

All *p*-values were adjusted for multiple testing using the false discovery rate (FDR) method, with FDR ≤ 0.05 considered statistically significant.

Although the analysis of clinical parameters has been reported previously [26], it was repeated here using the same statistical model but excluding the orthogonal noise term. This ensured methodological consistency, which was essential for the downstream integration of clinical, metabolomic, and lipidomic data.

Marginal means for each obesity × asthma group were extracted and visualized using the emmeans R package (version 1.8.4.1) [27]. Enrichment analysis of predefined metabolite and lipid classes among nominally significant features from the univariate models was performed using Fisher’s exact test, followed by FDR correction for multiple testing.

#### 2.8.3. Parsimonious Multivariate Predictive Models

Lasso regression with a binomial response was performed using the R package GLMNET (version 4.1.6) [28] on all features, including clinical, metabolomic, and lipidomic variables, after rank transformation and correction for confounders. Separate parsimonious predictive models of asthma were constructed for the OO and NW groups. Tenfold cross-validation was applied with the lambda parameter set to “1se.” Model performance was evaluated using receiver operating characteristic (ROC) curve analysis to assess sensitivity and specificity, implemented with the R package pROC (version 1.18.0) [29].

#### 2.8.4. Comparison with the QBB Adult Cohort

The QBB cohort has been described in detail previously [30]. Briefly, the QBB database includes comprehensive phenotypic profiles of Qatari nationals and long-term residents (≥15 years in Qatar) aged 18 years and older. It contains extensive baseline sociodemographic, clinical, and behavioral data, along with a wide range of biomarkers and comorbidities, including asthma. Serum samples from approximately 3000 QBB participants underwent untargeted metabolomics analysis following established protocols [31], generating around 1000 metabolite and lipid measurements.

Metabolite profiling was conducted using a Thermo Scientific Q-Exactive high-resolution/accurate-mass spectrometer (Thermo Fisher Scientific, Waltham, MA, USA) equipped with a heated electrospray ionization (HESI-II) source and an Orbitrap mass analyzer operating at 35,000 resolution. The mass spectrometer was coupled to a Waters ACQUITY ultra-performance liquid chromatography (UPLC) system (Waters Corporation, Milford, MA, USA). Full procedural details are available in earlier publications [31]. Metabolites were identified by matching against a library of more than 3300 pure standards and categorized by their biological source. Internal standards and QC measures have been described previously [32]. Briefly, stable isotope-labelled compounds were used to control for variability in sample preparation and instrument performance, while QC samples were analyzed throughout to monitor stability and reproducibility over time. Pre-analytical sample management, including collection, storage, and preparation, followed standardized protocols to minimize variability and preserve integrity. Because of the cross-sectional design, asthma-related lung function parameters were not available in the QBB dataset.

QBB participants were excluded if they were smokers, had a history of emphysema or chronic bronchitis, or lacked a confirmed asthma status. For consistency with the SOAP study design, the remaining 1069 QBB subjects were classified into four groups based on BMI and self-reported asthma status: NW_A (*n* = 60), NW (*n* = 602), OO_A (*n* = 41), and OO (*n* = 366), using a BMI cutoff of 25. QBB data were normalized using the rank-based inverse normal transformation across the dataset, and a linear model similar to that used with the SOAP cohort (incorporating confounders and an obesity interaction term) was applied to identify associations with asthma and BMI. Medication data was unavailable and not corrected for in the model. Meta-analysis and corresponding forest plots were generated using the R package metafor (version 4.6.0) [33] using a random-effects model. Enrichment analyses were conducted as described for the SOAP cohort. Mapping of metabolite and lipid species between the SOAP and QBB datasets was performed using MetaboAnalyst (version 5.0) [34] via HMDB and KEGG identifiers, supplemented by manual curation.

#### 2.8.5. Partial Correlation Network Analysis

All rank-transformed features, including clinical, metabolomic, and lipidomic variables, were analyzed using partial correlation based on Gaussian Graphical Models (GGM), implemented in the R package ggm (version 2.5.2) [35]. Significant partial correlations, after FDR-based multiple testing correction, representing associations between clinical traits and metabolites/lipids as well as cross-connections among metabolites and lipids, were selected. These were then imported into the open-source software platform Cytoscape (version 3.9.1) for network analysis and visualization [36].

#### 2.8.6. Stratification of BMI/Asthma Modulatory Effects by Gender

To further characterize gender-specific associations between obesity and asthma, we modified the linear model, described in Section 2.8.2, by including gender (sex) in the interaction term instead of treating it as a confounder:

Analyte~age + orthogonal_comp + medication_class1 + … + medication_class6 + obesity × bmi + obesity × asthma × sex

Multiple testing correction and downstream enrichment analysis were performed as previously described. However, we note the lack of statistical power for this analysis since stratification by gender was not the primary objective of this study.

## 3. Results

The SOAP cohort used in this study was predominantly of Middle Eastern and North African origin (93%), with most participants holding Qatari nationality (60%) and additional groups from other Arab countries, including Egypt (14%), Sudan (8%) and other Gulf states (4%). A smaller proportion of children were of South Asian (Indian), European (Dutch and French) origin. The cohort featured more males (*n* = 223) than females (*n* = 138), with male sex further enriched amongst asthmatic participants (Table 1). An overview of the study design and analysis pipeline is presented in (Figure 1A).

The following sections provide a detailed account of the results, organized by study objective.

### 3.1. Clinical, Physiological, and Inflammatory Characteristics Across BMI and Asthma Groups

As reported previously [19,26], children in the OO group showed expectedly higher basal metabolic rate, and blood pressure compared with their normal-weight peers, irrespective of asthma status (Supplementary Table S1). A significantly higher proportion of males than females was observed among asthmatic children, independent of obesity (Supplementary Table S1). Within the OO group, OO_A children had, on average, slightly lower BMI, weight, body fat, and metabolic rate than the overall OO mean (Supplementary Table S1). As such, sex and BMI were identified as potential confounders and were adjusted for in all subsequent analyses (see Section 2). In our cohort, the majority of participants had mild asthma; approximately 75% had an FEV_1_ ≥ 80%, with very few children meeting criteria for moderate or severe obstruction.

In both the OO and NW groups, we detected several clinical features that were significantly elevated in association with asthma after correction for confounders. These included a higher incidence of rhinitis and allergies, as well as elevated inflammatory markers such as FeNO and eosinophil count. This inflammatory profile was accompanied by reduced lung function, evidenced by lower FEV1/FVC and FEF 25–75% values, observed consistently in both OO and NW groups (Table 2, Supplementary Table S2).

We also noted group-specific effects. In the NW group, asthma was associated with increased sodium-to-potassium ratio and higher levels of leptin and interleukins IL-5, IL-17A, IL-22, and IL-33, as previously reported [26] (Table 1 and Figure 1B). By contrast, changes specific to the OO group were less pronounced and included a modest increase in RV/TLC ratio at nominal significance prior to multiple testing correction (Table 2 and Figure 1B). Additional, less prominent changes observed in both NW and OO groups at nominal significance are summarized in Supplementary Table S2. Together, these findings confirm distinct clinical and inflammatory profiles associated with asthma across BMI groups, providing the foundation for subsequent metabolomic and lipidomic analyses.

### 3.2. Metabolomic and Lipidomic Differences Across BMI and Asthma Groups

**Figure 2:**
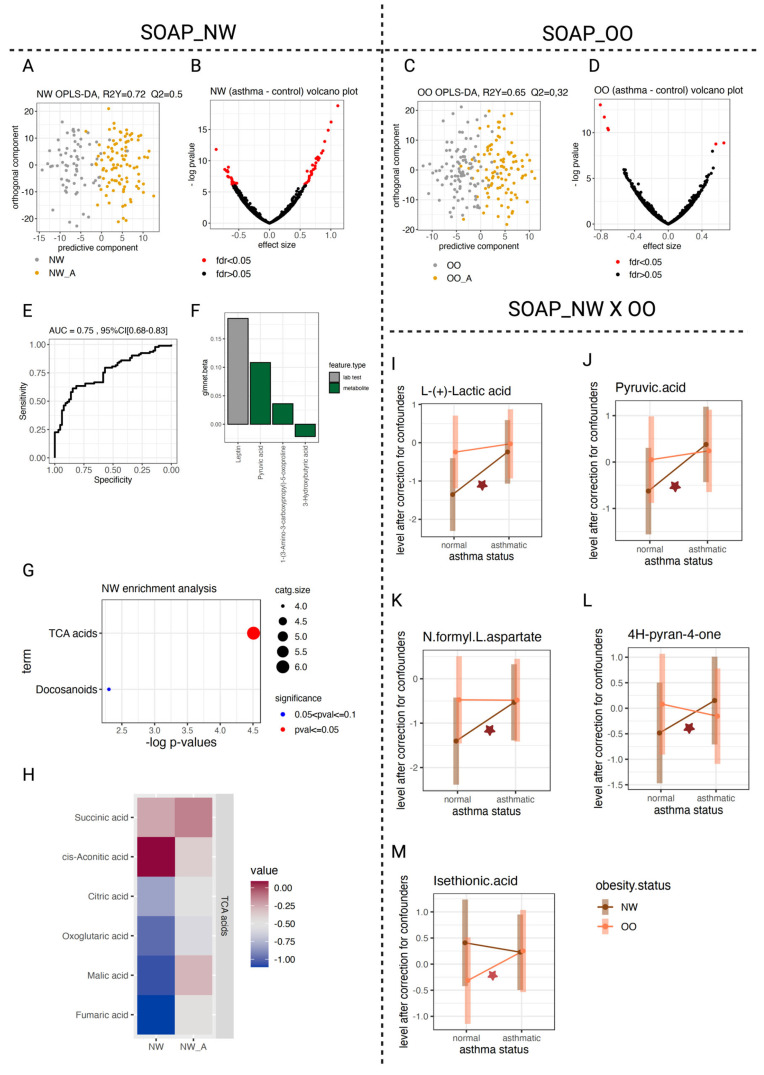
Metabolomic association with asthma in NW and OO. (**A**,**C**) OPLS-DA plots showing separation between asthmatic and non-asthmatic children in NW and OO groups, respectively, based on the predictive component (X-axis) as opposed to the orthogonal noise component (Y-axis). (**B**,**D**) Volcano plots from univariate analyses with correction for confounders, highlighting metabolites significantly associated with asthma in NW and OO. (**E**,**F**) Lasso regression analysis in NW showing ROC curve performance and regression coefficients of selected predictive features. (**G**) Functional enrichment analysis of nominally significant metabolites showing overrepresentation of TCA cycle metabolites in NW (no enriched functions were identified in OO). (**H**) Heatmap showing increased mean levels of TCA intermediates in NW after confounder correction. (**I**–**M**) Examples of significant asthma X obesity interaction effects showing BMI group–specific metabolite changes. The red star indicates nominal significance at *p* value ≤ 0.05.

**Figure 3:**
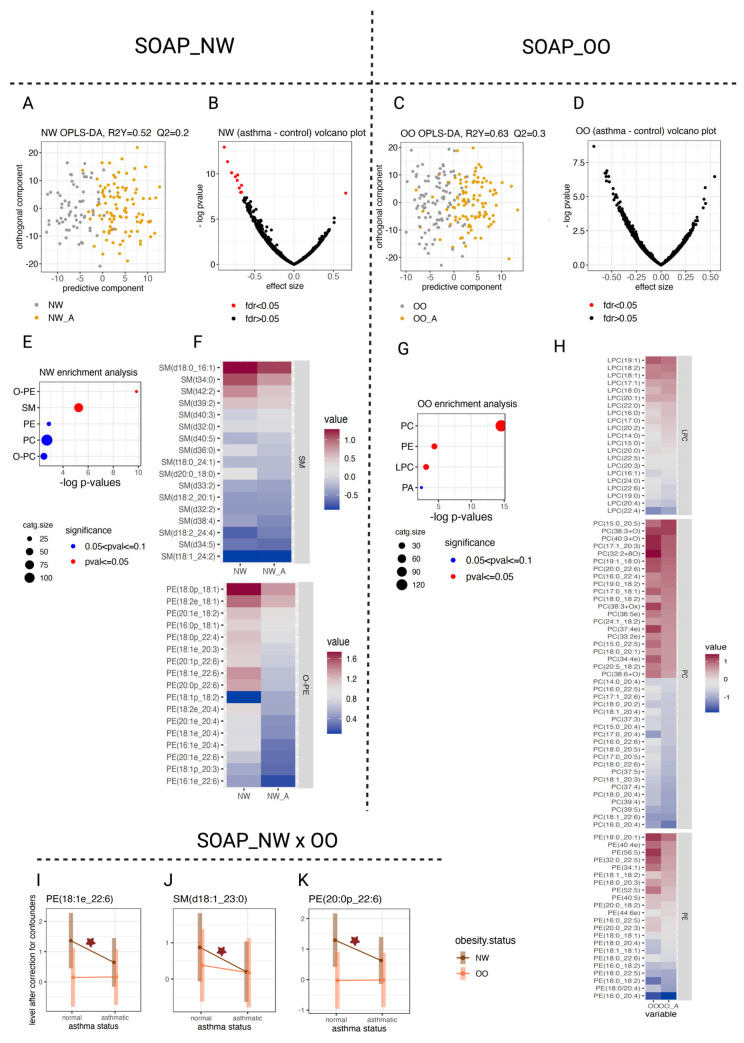
Lipidomic associations with asthma in NW and OO. (**A**,**C**) OPLS-DA plots showing separation between asthmatic and non-asthmatic children in NW and OO groups, respectively. (**B**,**D**) Volcano plots from univariate analyses with correction for confounders, showing lipids significantly associated with asthma in NW and OO. (**E**,**G**) Functional enrichment analysis showing overrepresentation of ether-linked phosphatidylethanolamines (O-PEs) and sphingomyelins (SMs) in NW, versus phosphatidylcholines (PCs), phosphatidylethanolamines (PEs), and lysophosphatidylcholines (LPCs) in OO. (**F**) Heatmap showing alterations in SM and PE levels in NW asthmatics. (**H**) Heatmap showing changes in LPC, PC, and PE levels in OO asthmatics. (**I**–**K**) Examples of significant asthma × obesity interaction effects showing BMI group–specific lipid changes. The red star indicates nominal significance at *p* value ≤ 0.05.

To explore metabolic and lipidomic alterations associated with asthma, we first compared global molecular profiles across BMI groups using multivariate analysis followed by univariate approaches. We detected a clear separation between asthmatic and non-asthmatic children in the multivariate metabolomic/lipidomic profiles, with the NW group showing a stronger distinction (R^2^Y = 0.72/0.52) than the OO group (R^2^Y = 0.65/0.63), as revealed by OPLS-DA analysis (Figure 2A,C and Figure 3A,C). Cross-validation confirmed the robustness of these models, yielding a significant Q^2^ (>0.5) for the NW metabolomics data (Figure 2A). Univariate analysis further indicated that the extent of asthma-related metabolic and lipidomic changes was greater in NW than in OO (FDR ≤ 0.05) (Figure 2B,D and Figure 3B,D; Table 3 and Table 4).

We next examined individual metabolite and lipid contributions to asthma in a group-specific manner. In NW children, we observed increased levels of lactate, pyruvate, N-formyl-L-aspartate and 4H-pyran-4-one (Figure 2I–L), along with reduced levels of ether-linked phosphatidylethanolamines (O-PE) and sphingomyelins (SM) (Figure 3I–K). In contrast, OO children showed a selective upregulation of isethionic acid (Figure 2M; Table 3 and Table 4; Supplementary Tables S3 and S4).

When we applied Lasso regression, leptin emerged as the strongest predictor of asthma in NW. Three additional metabolites—pyruvic acid, 1-(3-amino-3-carboxypropyl)-5-oxoproline, and 3-hydroxybutyric acid—contributed marginally to the model, which achieved strong predictive power (AUC = 0.75, *p* ≤ 10^−16^) (Figure 2E,F). No comparable predictive model was obtained for the OO group.

Consistent with the univariate findings, our enrichment analysis revealed distinct biological signatures across BMI categories. In NW, asthma was associated with heightened TCA cycle activity (Figure 2G,H), reflected by significant or borderline increases in pyruvate, lactate, malate, and fumarate (Figure 2H–J; Table 3). We also found a marked decrease in O-PEs and altered SM metabolism (Figure 3E,F,I–K; Table 4). Conversely, in OO, asthma induced more subtle lipidomic remodeling, mainly involving phosphatidylethanolamines (PE), phosphatidylcholines (PC), and lysophosphatidylcholines (LPC) (Figure 3G,H; Table 4). No metabolite class reached significant enrichment among the top regulated features in OO. Together, these results reveal BMI-dependent molecular patterns in pediatric asthma, characterized by TCA activation and altered sphingolipid metabolism in NW versus subtle phospholipid dysregulation in OO.

### 3.3. Comparison of Pediatric and Adult Metabolic and Lipidomic Signatures of Asthma

To extend our findings beyond the pediatric cohort, we next investigated asthma in the adult population using data from the QBB cohort. Because of the substantial age differences between cohorts, we did not treat QBB as a replication cohort but rather as a means to explore potential mechanistic overlap between adult and pediatric asthma. To ensure comparability, we classified QBB participants into the same four groups (NW, NW_A, OO, and OO_A) and applied equivalent data transformation and analysis procedures as in the SOAP study (see Section 2).

We noted a higher incidence of asthma among OO adults compared with NW adults, although this difference did not reach statistical significance (asthma frequency = 0.112 vs. 0.099 for OO and NW, respectively; *p* = 0.3). Asthma prevalence was significantly higher among NW females than males (*p* = 0.009), and among OO participants it increased with age (Supplementary Table S5). No significant multivariate predictive components were identified by OPLS-DA in either NW or OO. Similarly, univariate comparisons between asthmatics and controls in both BMI groups revealed only subtle metabolite level changes relative to SOAP, likely reflecting the population-based nature of the QBB cohort and the less stringently defined, self-reported asthma phenotype.

**Figure 4:**
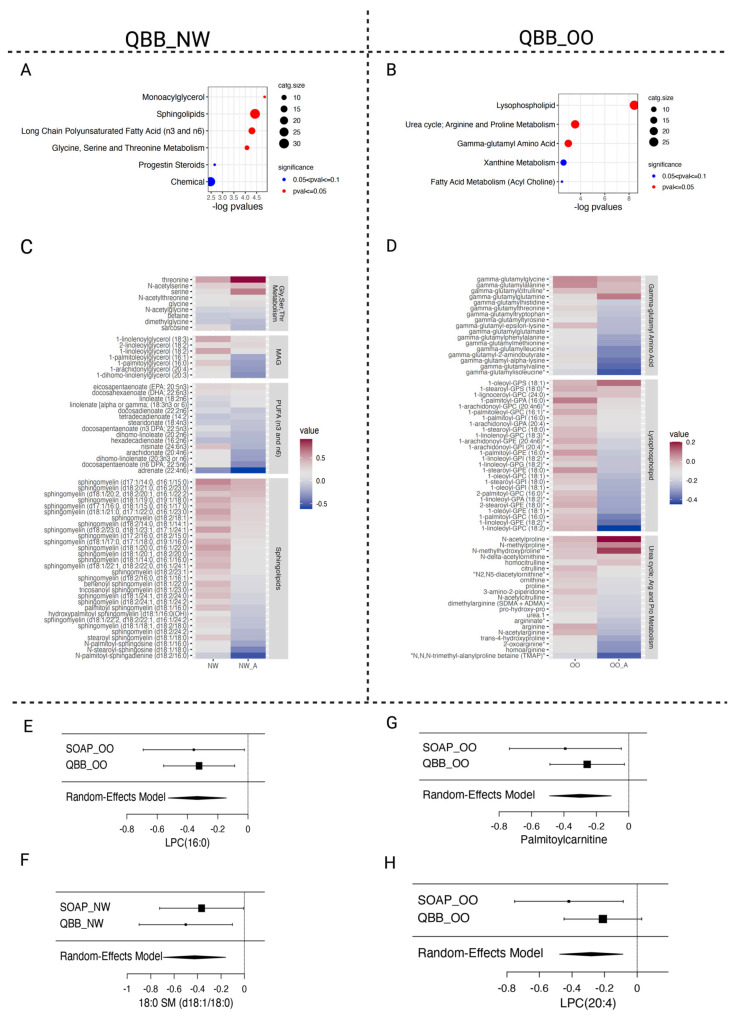
Asthma-related metabolomic and lipidomic effects in the adult QBB cohort. (**A**,**B**) Functional enrichment analyses in QBB_NW and QBB_OO replicating sphingomyelin and LPC dynamics observed in the pediatric SOAP cohort. (**C**,**D**) Heatmaps showing reduced mean levels of SMs, monoacylglycerols (MAGs), LPCs, urea cycle intermediates, and γ-glutamyl amino acids in adult NW and OO asthmatics. (**E**–**H**) Meta-analysis, using a random-effects model, confirming consistent asthma-associated decreases in SM, PC, and LPC species across the SOAP and QBB cohorts. For figure (**C**,**D**)—No star represents confirmed metabolite identity, * represents high-confidence but not fully confirmed identity, and **—represents metabolites with tentative/lower-confidence structural identity.

Although few global differences were detected, we next explored whether any biological pathways were consistently affected across BMI groups. Functional enrichment analysis of the nominally significant metabolites revealed several biologically relevant trends. In NW adults, we found a differential abundance of monoacylglycerols (MAG), sphingolipids, long-chain polyunsaturated fatty acids (PUFA), and pathways involving glycine, serine, and threonine metabolism (Figure 4A,C). In OO adults, asthma was characterized primarily by alterations in LPC metabolism, together with enrichment of the urea cycle, arginine and proline metabolism, and γ-glutamyl amino acid pathways (Figure 4B,D).

When we compared these findings with those from the SOAP study, we observed a clear overlap in the relevance of SMs and LPCs to asthma pathogenesis—SMs predominating in NW and LPCs in OO—in both adult and pediatric populations (Figure 3F,H and Figure 4C,D). Meta-analysis of the SM and PC/LPC species that reached nominal significance in the SOAP cohort confirmed a consistent pattern across both datasets, showing significant decreases in 18:0 SM (d18:1/18:0), palmitoylcarnitine, and LPC (20:4) in association with asthma in both children and adults (Figure 4E–H; Table 5). Together, these findings suggest that key lipid pathways, particularly those involving sphingomyelins and lysophosphatidylcholines, contribute to asthma pathophysiology across ages and BMI categories.

### 3.4. Integration of Clinical, Metabolic, and Lipid Correlates of Asthma via GGM Analysis

**Figure 5:**
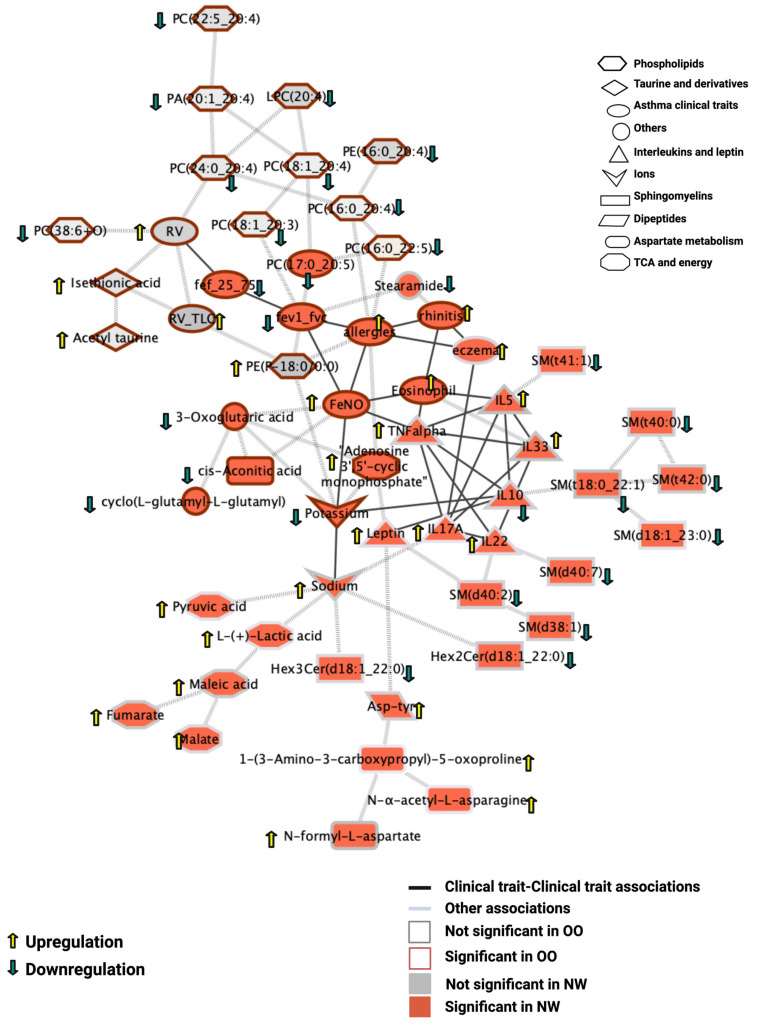
Integrative partial correlation network of asthma-associated features. Network graph showing significant partial correlations between clinical traits, lung function measures, blood parameters, and metabolomic/lipidomic features after correction for all other variables. Connections indicate direct associations distinguishing asthma effects in NW and OO. The network highlights group-specific and shared mechanisms, including links between sphingomyelins and interleukins in NW, phospholipid decline and antioxidant upregulation in OO, and clinical features that are common in both groups, such as reduced FEF 25–75% and FEV1, and elevated FeNO and eosinophils.

To uncover coordinated relationships among clinical, metabolic, and lipidomic features associated with asthma, we applied partial correlation analysis based on GGM. This approach identifies direct connections after accounting for indirect association, reflecting general association relationships rather than causal effects. The resulting network (Figure 5) provides an integrated view of asthma-related interactions, revealing coordinated shifts between clinical traits and metabolic or lipidomic pathways.

In NW children, we observed that changes in SM levels were directly connected to interleukin concentrations, suggesting an interaction between lipid signaling and inflammatory activity. In parallel, increased levels of TCA cycle intermediates and aspartate-derived energy mediators correlated with elevated sodium and reduced potassium levels, suggesting a potential imbalance in metabolic–electrolyte regulation. Leptin emerged as a central node within this network, linking inflammatory and metabolic axes and potentially influencing both. By contrast, the OO network showed distinct patterns reflecting the metabolic stress of obesity.

In the OO group, higher residual air volumes (RV and RV/TLC) seem to be associated with decreased phospholipid levels, accompanied by an upregulation of antioxidant metabolites such as acetyltaurine, the taurine derivative isethionic acid, and the plasmalogen 1-(1Z-octadecenyl)-sn-glycero-3-phospho-(N-palmitoyl)ethanolamine (PE(P-18:0/0:0)). These metabolites may provide protection against oxidative stress associated with obesity-related inflammation.

Across both BMI groups, the network also captured shared asthma effects, including reduced forced expiratory flow and volume (FEF 25–75% and FEV1) and the co-occurrence of related inflammatory conditions such as eczema, rhinitis, and allergies. Notably, elevated FeNO and eosinophil counts in both groups were linked to depletion of the antioxidant cyclo-L-glutamyl-L-glutamyl and the TCA intermediate cis-aconitate (Figure 5), highlighting a common oxidative–inflammatory signature in pediatric asthma. Together, the GGM analysis highlights how metabolic, inflammatory, and functional markers converge to define BMI-specific and shared mechanisms of pediatric asthma.

### 3.5. Stratification of Effects by Gender

**Figure 6:**
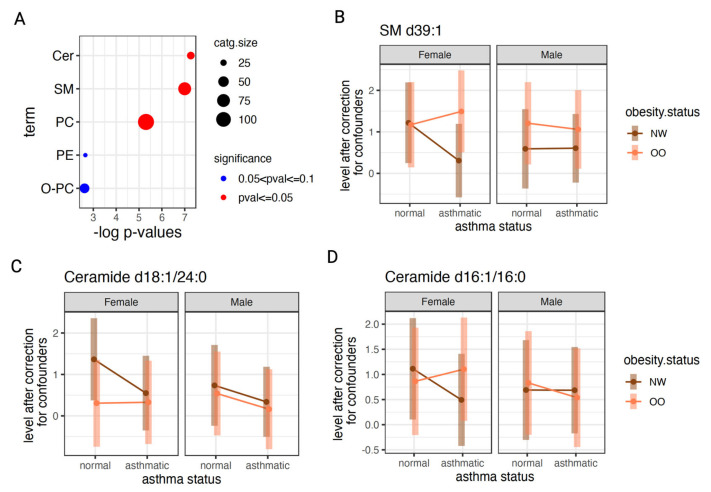
Stratification of obesity-related asthma effects by gender. (**A**) Enrichment analysis showing significantly over-represented ceramides, sphingomyelins and phosphocholines. (**B**–**D**) Example ceramide and sphingomyelin species showing nominally significant differential effects in OO versus NW with asthma, observed in females only.

To explore whether obesity-related asthma effects differed by gender, we extended the linear model to include a three-way interaction term (obesity × asthma × sex), as described in Section 2.8.6. In these sex-stratified models, no individual metabolite or lipid reached statistical significance after correction for multiple testing, which is consistent with limited power given that sex-specific effects were not a primary design objective. Nevertheless, pathway-level enrichment based on nominal lipid effects highlighted a coherent pattern involving ceramides, sphingomyelins, and phosphatidylcholines (Figure 6A). Representative interaction plots for sphingomyelin d39:1 and ceramides d18:1/24:0 and d16:1/16:0 (Figure 6B–D) illustrate that obesity-associated asthma is accompanied by more pronounced shifts in these lipids among females than males. These preliminary results require confirmation in a larger cohort to determine whether modulation of sphingolipid and ceramide metabolism is a major contributor to obesity-related asthma, particularly in females.

## 4. Discussion

Obesity and asthma are two major public health challenges that continue to rise in prevalence, particularly among children. Numerous studies have established a positive association between higher BMI and an increased risk of asthma development and exacerbations in the pediatric population, with meta-analyses reporting 20–40% higher risk in overweight and obese children compared with their normal-weight peers [4,37,38,39]. The mechanisms underlying this relationship are multifactorial, involving both mechanical and inflammatory pathways [3,9,40,41]. While obesity is linked to asthma severity and poorer control, the molecular mechanisms connecting metabolic dysfunction and airway pathology in children remain poorly defined. Several metabolomics-based studies have explored the interplay between obesity and asthma [13,42,43], and identified alterations in lipid and amino acid metabolism, oxidative stress markers, or sphingolipid pathways in obesity-related asthma. Yet, they were limited by small sample sizes, incomplete control for confounders (e.g., medication), or lack of integration across omic layers [13,42,43].

### 4.1. Study Novelty

Here, we established a large, well-characterized multi-omics pediatric cohort and implemented a design that directly compares asthmatic children to BMI-matched controls. The validity of our data is supported by expected trends in key clinical parameters, including reduced FEF 25–75% and FEV1/FVC ratios, and increased LCI, FeNO, and eosinophil counts in asthmatic children overall. We also observed higher rates of atopic comorbidities such as rhinitis, allergies, and eczema, consistent with typical asthma-associated immune profiles, irrespective of BMI group [44]. After rigorous statistical corrections for confounding factors, we derived several novel insights into the molecular mechanisms underlying pediatric asthma. Most notably, we identified a central role for leptin in NW asthma, contrary to prior assumptions that leptin dysregulation is confined to obesity-associated asthma [45,46]. Furthermore, in this study, leptin levels were similarly elevated in obese asthmatics and their BMI-matched controls, consistent with obesity-driven leptin resistance and suggesting that leptin levels per se may no longer discriminate asthma status in this group. Further, extending previous observations that leptin is more informative as a risk factor than as a disease classifier in established obesity [47,48].

We also uncovered a key role for SM metabolism in NW asthma, a pathway typically linked to obesity-associated asthma and to asthma susceptibility through 17q21-related sphingolipid endotypes. Recent pediatric work has shown that different sphingolipid subclasses relate differentially to asthma. While ceramides preferentially associate with risk factors such as adiposity and reduced lung function, sphingolipids involved in recycling and catabolic pathways show stronger links to asthma phenotypes and worsened lung function [49]. Our finding that SM metabolism is most perturbed in NW asthma, while phospholipid metabolism (and lysophospholipid production) is more relevant in OO asthma, aligns with this class-specific view and suggests that distinct sphingolipid modules contribute to asthma pathophysiology across BMI strata. Importantly, these BMI-specific shifts in sphingolipid and lysophospholipid metabolism observed in children were mirrored in the adult QBB cohort, underscoring their broader biological relevance in asthma.

By integrating metabolomic and lipidomic data with detailed clinical phenotyping, we established direct physiological links between SM degradation, interleukin activation, and leptin signaling in NW asthma, as well as between phospholipid remodeling, antioxidant metabolism, and increased residual lung volume in OO asthma. Previous multi-omics work in obese allergic asthmatic children reported higher leptin and altered short-chain fatty acid and microbiota signatures, and a recent narrative review highlighted ketone bodies, amino acid metabolism, and oxidative stress pathways as key components of obesity-related asthma [43,47]. Our results extend these observations by connecting specific metabolic pathways—such as taurine derivatives, TCA cycle intermediates, and SM turnover—to quantitative lung function measures (RV/TLC, LCI) and inflammatory cytokines, thereby providing a systems-level view of asthma heterogeneity across BMI groups.

### 4.2. Asthma in Obese

When stratified by BMI, several clinical and pulmonary features were differentially affected by asthma. Notably, leptin, IL-5, and IL-33 were altered in NW children, whereas RV/TLC ratio increased among OO children, in agreement with previous findings from the same cohort [26].

We also identified distinct metabolic and physiological signatures of asthma in OO compared with NW children. Most notably, OO asthmatics exhibited a higher degree of air trapping than their NW counterparts, as reflected by increased RV/TLC ratios, indicating more pronounced airway obstruction. We also observed a decline in phospholipid levels in OO, which may result from oxidative stress-induced degradation processes that generate lysophospholipids—bioactive molecules known to contribute to airway remodeling and fibrosis [50]. Interestingly, a similar phospholipid depletion pattern was detected in adult OO asthmatics from the QBB cohort, reinforcing the relevance of this mechanism across age groups. Further, these results complement reports in adults with obesity-related asthma where multiple lipid pathways, including long-chain fatty acids and phospholipids, are dysregulated [43].

Our findings further support a role for oxidative stress in OO asthma, likely driven by chronic obesity-related inflammation. This interpretation is consistent with the marked upregulation of the taurine derivative isethionic acid and its precursor acetyltaurine in OO asthmatics, but not in NW individuals. This resonates with previous demonstrations that oxidative stress markers such as malondialdehyde and reduced glutathione are altered in children with asthma and in obesity-related asthma phenotypes [51,52]. Although taurine itself was not measured in this study, these closely related metabolites may retain its antioxidant and osmoprotective properties, helping to counteract oxidative stress in the obese state. Moreover, the stronger reduction in the TCA cycle intermediate cis-aconitic acid and the antioxidant cyclo-L-glutamyl-L-glutamyl in OO suggests a shift in cellular redox balance, likely reflecting a diversion of TCA intermediates toward sustaining enhanced antioxidant defenses. Reviews on obesity-related asthma propose oxidative stress as one mechanistic link by which obesity worsens or modifies asthma, but they also emphasize the evidence is largely associative and mechanistic rather than from longitudinal human causal studies [43].

### 4.3. Asthma in Normal Weights

The metabolic and lipidomic signature of asthma in NW children differed markedly from that in OO. First, we observed a distinct increase in TCA cycle flux, reflected by elevated fumarate and malate levels, alongside accumulation of pyruvate and aspartate precursors and an increased sodium-to-potassium (Na^+^/K^+^) ratio. While previous metabolomic studies have identified TCA cycle intermediates and amino acid changes in non-obese childhood asthma, our data uniquely connect these shifts to Na^+^/K_+_ homeostasis and leptin-driven inflammatory pathways. Second, we identified broad alterations in SM levels, which correlated with elevated inflammatory interleukins, most notably IL-5, IL-33, and TNF-α. Both GGM partial correlation analysis and Lasso regression positioned leptin as a central regulator of asthma pathology in NW children. Leptin is known to trigger histamine release [53], which modulates cyclic AMP (cAMP) levels [54] and activates the Na^+^/K^+^ pump. This activation promotes autophagy in adipose tissue and lung fibroblasts [55], leading to the degradation of proteins and membrane lipids, including SMs, and thereby releasing proinflammatory mediators [56,57]. These processes recruit immune cells such as macrophages and monocytes, which further amplify inflammation through TNF-α and interleukin release [57]. The Na^+^/K^+^ pump and inflammatory processes are energy demanding, explaining the increased TCA cycle flux and amino acid shuttling observed in NW asthma.

Leptin also exerts direct proinflammatory effects by stimulating cytokine production and activating inflammatory pathways in bronchial epithelial cells [45]. Interestingly, both leptin and SM metabolism have been implicated in microglia–neuron communication, suggesting that metabolic signaling may influence immune–neuronal crosstalk. The neuroimmune axis is increasingly recognized as a central component of asthma pathophysiology [58,59], and our findings further support its involvement in pediatric asthma.

### 4.4. Gender-Specific Effects

Our gender-stratified analyses further implicate sphingolipids as central mediators of the asthma–obesity axis, with analyte-specific patterns for Cer d18:1/24:0, Cer d16:1/16:0 and SM d39:1. Ceramides were among the most significantly enriched lipid classes in our interaction analysis, consistent with prior work showing that obesity-related asthma is characterized by a shift toward long-chain ceramide accumulation and higher circulating ceramide levels in uncontrolled asthma. Long-chain and very-long-chain species such as C16:0 and C24:0 are repeatedly linked to obesity, diabetes and cardiometabolic disease, and C16:0 ceramide in particular has been implicated in obesity-related inflammation and insulin resistance, in line with the obesity-dependent changes that we observe for Cer d16:1/16:0 [60]. Importantly, circulating ceramide profiles are sexually dimorphic, with males exhibiting differential concentrations of ceramides including Cer16:0–Cer24:0, which supports the sex-specific modulation we detect for Cer d18:1/24:0 and Cer d16:1/16:0 [61]. Sphingomyelins, including longer-chain species analogous to SM d39:1, have likewise been associated with asthma and obesity in human metabolomics studies and experimental work shows that perturbing sphingolipid synthesis enhances airway hyperresponsiveness. Together with recent evidence that obesity and sex jointly shape allergic airway inflammation in vivo, these data support our observation that ceramide and sphingomyelin remodeling along the asthma–obesity axis is potentially further modulated by sex, providing a mechanistic context for the gender-specific lipid signatures identified in our cohort [62,63].

### 4.5. Study Limitations

Our study is primarily limited by having a cross-sectional design, which prevents us from establishing a cause–effect relationship. Furthermore, our study used plasma samples to analyze metabolomic and lipidomic differences, with further confirmation, using samples from the airway compartment, needed to confirm our findings.

Our analysis involved multiple high-dimensional modeling steps, which carries an inherent risk of both false-positive and false-negative findings. Although cross-validation and penalization with Lasso reduce overfitting on average, they do not guarantee correct variable selection in a specific dataset, and selected predictors may include false positives driven by sampling variability, especially when the effective sample size is modest relative to the number of candidates. Conversely, true associations may be missed if they are modest in magnitude or highly correlated with other features that are retained instead. We partially mitigated these issues by (i) restricting formal inference to a limited set of pre-specified contrasts, (ii) using regularization tuned by cross-validation, and (iii) focusing on biological coherence and consistency across complementary analyses as well as datasets through meta-analysis. Nevertheless, our results should be viewed as hypothesis-generating, and external validation and replication in independent cohorts will be essential to confirm the identified metabolite and pathway signals.

## 5. Conclusions

In summary, our study provides a comprehensive metabolomic framework for understanding the interplay between asthma and obesity. By integrating multi-omics data with detailed phenotyping, we identified inflammation as the predominant mechanism driving asthma in NW children, potentially mediated by elevated leptin levels, and found that oxidative stress features prominently in obesity-related asthma. Given the cross-sectional design, these patterns should be interpreted as associations rather than definitive causal pathways, although they are consistent with models in which long-term obesity increases systemic and airway oxidative stress that may, in turn, exacerbate asthma in susceptible children. These insights refine current models of asthma heterogeneity and may guide precision medicine approaches to disease management in the pediatric population. Now, further research, including longitudinal and well-controlled interventional studies, will be essential to confirm these mechanisms and translate them into clinically actionable strategies for asthma management. Additionally, future studies examining how the identified mechanisms influence treatment responses, and whether they can be reversed through weight management or biologic therapies in obesity-associated asthma, will be of clinical significance.

## Figures and Tables

**Table 1:** General characteristics of study participants.

	(NW_A) (*n* = 95)	(NW) (*n* = 67)	(OO_A) (*n* = 99)	(OO) (*n* = 100)	*p* Value (NW_A × NW)	*p* Value (OO_A × OO)
Gender	M: 70, F: 25	M: 36, F: 31	M: 72, F: 27	M: 45, F: 55	1.17 × 10^−2^	9.29 × 10^−5^
Age (year)	10 (8–13.5) 11 ± 3.14	10 (8–13) 11 ± 3.13	12 (9–15) 12 ± 2.87	13 (10.5–14.5) 12 ± 3.34	6.24 × 10^−1^	5.57 × 10^−1^
BMI percentile	45 (21–69)	38.5 (17–58.75)	97 (94–99)	98.5 (97–100)	7.50 × 10^−3^	<1 × 10^−4^

BMI_percentile, which is right-skewed, is presented as median (IQR). Age, showing no major distributional skewness, is given as median (IQR) and mean ± sd. The Chi-square test was used with the categorical variable gender, whilst the *t*-test was used with gender and BMI_percentile post rank-normal transformation. Abbreviations: NW, Normal weight without asthma; NW_A, Normal weight with asthma; OO, Overweight/obesity without asthma; OO_A, Overweight/obesity with asthma.

**Table 2:** Phenotype association with asthma in NW and OO with correction for confounders.

Phenotype	NW_A vs. NW			OO_A vs. OO			NW × OO *		
	Beta	*p* Value	FDR	Beta	*p* Value	FDR	Beta	*p* Value	FDR
Leptin	0.858	2.14 × 10^−10^	2.16 × 10^−8^	0.132	2.70 × 10^−1^	6.20 × 10^−1^	−0.726	3.20 × 10^−6^	3.04 × 10^−4^
Rhinitis	0.404	2.32 × 10^−7^	1.17 × 10^−5^	0.392	3.13 × 10^−8^	3.16 × 10^−6^	−0.012	8.97 × 10^−1^	9.34 × 10^−1^
Sodium	0.849	1.55 × 10^−5^	4.15 × 10^−4^	0.031	8.57 × 10^−1^	9.39 × 10^−1^	−0.818	3.06 × 10^−4^	1.45 × 10^−2^
FEV1/FVC	−0.771	1.64 × 10^−5^	4.15 × 10^−4^	−0.633	8.74 × 10^−5^	3.12 × 10^−3^	0.138	5.07 × 10^−1^	8.61 × 10^−1^
FeNO	0.784	2.59 × 10^−5^	5.22 × 10^−4^	0.661	9.27 × 10^−5^	3.12 × 10^−3^	−0.123	5.73 × 10^−1^	8.89 × 10^−1^
Potassium	−0.778	9.70 × 10^−5^	1.63 × 10^−3^	−0.391	2.67 × 10^−2^	1.59 × 10^−1^	0.388	9.05 × 10^−2^	4.97 × 10^−1^
Eosinophil	0.683	1.40 × 10^−4^	2.02 × 10^−3^	0.481	2.94 × 10^−3^	4.95 × 10^−2^	−0.202	3.36 × 10^−1^	7.58 × 10^−1^
IL5	0.685	2.25 × 10^−4^	2.82 × 10^−3^	−0.017	9.19 × 10^−1^	9.47 × 10^−1^	−0.702	1.30 × 10^−3^	3.08 × 10^−2^
IL33	0.661	2.51 × 10^−4^	2.82 × 10^−3^	−0.033	8.40 × 10^−1^	9.39 × 10^−1^	−0.695	1.08 × 10^−3^	3.08 × 10^−2^
Eczema	0.255	5.05 × 10^−4^	4.81 × 10^−3^	0.051	4.42 × 10^−1^	7.70 × 10^−1^	−0.205	1.73 × 10^−2^	2.74 × 10^−1^
FEF 25–75%	−0.628	5.51 × 10^−4^	4.81 × 10^−3^	−0.513	1.75 × 10^−3^	3.87 × 10^−2^	0.114	5.89 × 10^−1^	8.89 × 10^−1^
Allergies	0.258	5.72 × 10^−4^	4.81 × 10^−3^	0.210	1.92 × 10^−3^	3.87 × 10^−2^	−0.048	5.83 × 10^−1^	8.89 × 10^−1^
IL17A	0.474	5.02 × 10^−3^	3.90 × 10^−2^	0.212	1.69 × 10^−1^	4.86 × 10^−1^	−0.262	1.86 × 10^−1^	6.11 × 10^−1^
IL22	0.480	6.88 × 10^−3^	4.96 × 10^−2^	0.147	3.63 × 10^−1^	7.22 × 10^−1^	−0.333	1.11 × 10^−1^	4.97 × 10^−1^
Histamine	1.191	1.12 × 10^−2^	7.56 × 10^−2^	−0.205	5.48 × 10^−1^	8.02 × 10^−1^	−1.396	1.43 × 10^−2^	2.71 × 10^−1^
IL13	0.308	9.26 × 10^−2^	2.91 × 10^−1^	−0.185	2.70 × 10^−1^	6.20 × 10^−1^	−0.493	2.26 × 10^−2^	3.07 × 10^−1^
RV/TLC	−0.029	8.77 × 10^−1^	9.24 × 10^−1^	0.401	1.41 × 10^−2^	1.07 × 10^−1^	0.429	4.70 × 10^−2^	4.97 × 10^−1^
SBP	0.278	9.45 × 10^−2^	2.91 × 10^−1^	−0.110	4.65 × 10^−1^	7.83 × 10^−1^	−0.387	4.79 × 10^−2^	4.97 × 10^−1^

* NW × OO is the interaction effect reflecting, if significant, differential response to asthma between the two groups, statistically given as (OO_A vs. OO)—(NW_A vs. NW). Only features with an FDR ≤ 0.05 in either NW or OO or an interaction effect NW × OO with a nominal *p* value ≤ 0.05 are shown. Abbreviations: FEF 25–75%, Forced expiratory flow at 25% and 75% of the pulmonary volume; FEV1, FeNO, Fractional exhaled nitric oxide; Forced expiratory volume in 1 s; FVC, Forced vital capacity; IL, Interleukin; NW, Normal weight without asthma; NW_A, Normal weight with asthma; OO, Overweight/obesity without asthma; OO_A, Overweight/obesity with asthma; RV, Residual volume; SBP, systolic blood pressure; TLC, Total lung capacity.

**Table 3:** Metabolomics association with asthma in NW and OO with correction for confounders.

	NW_A vs. NW			OO_A vs. OO			NW × OO *		
Metabolite	Effect Size	*p* Value	FDR	Effect Size	*p* Value	FDR	Effect Size	*p* Value	FDR
Lactic acid	1.11	6.85 × 10^−9^	8.21 × 10−^6^	2.14 × 10^−1^	2.07 × 10^−1^	7.86 × 10^−1^	−8.99 × 10^−1^	5.63 × 10^−5^	4.21 × 10^−2^
Pyruvic acid	1.00	9.25 × 10^−8^	5.54 × 10^−5^	1.90 × 10^−1^	2.54 × 10^−1^	7.94 × 10^−1^	−8.14 × 10^−1^	2.00 × 10^−4^	4.21 × 10^−2^
1-(3-Amino-3-carboxypropyl)-5-oxoproline	9.57 × 10^−1^	3.45 × 10^−7^	1.38 × 10^−4^	2.90 × 10^−1^	8.24 × 10^−2^	7.54 × 10^−1^	−6.67 × 10^−1^	2.25 × 10^−3^	1.60 × 10^−1^
Isethionate	−8.68 × 10^−1^	7.44 × 10^−6^	1.78 × 10^−3^	−1.82 × 10^−1^	2.93 × 10^−1^	8.08 × 10^−1^	6.86 × 10^−1^	2.41 × 10^−3^	1.60 × 10^−1^
N-Formyl-L-aspartate	8.72 × 10^−1^	9.25 × 10^−6^	1.85 × 10^−3^	−8.83 × 10^−3^	9.60 × 10^−1^	9.96 × 10^−1^	−8.81 × 10^−1^	1.33 × 10^−4^	4.21 × 10^−2^
Asp-Tyr	8.48 × 10^−1^	1.34 × 10^−5^	2.29 × 10^−3^	8.73 × 10^−2^	6.15 × 10^−1^	9.37 × 10^−1^	−7.61 × 10^−1^	8.45 × 10^−4^	1.26 × 10^−1^
Malic acid	7.79 × 10^−1^	2.81 × 10^−5^	3.18 × 10^−3^	1.47 × 10^−1^	3.76 × 10^−1^	8.29 × 10^−1^	−6.32 × 10^−1^	3.67 × 10^−3^	1.72 × 10^−1^
N-Acetyl-L-asparagine	8.10 × 10^−1^	3.03 × 10^−5^	3.18 × 10^−3^	2.90 × 10^−1^	9.47 × 10^−2^	7.64 × 10^−1^	−5.19 × 10^−1^	2.18 × 10^−2^	2.59 × 10^−1^
Gallic acid	7.44 × 10^−1^	1.04 × 10^−4^	7.76 × 10^−3^	1.53 × 10^−1^	3.73 × 10^−1^	8.29 × 10^−1^	−5.91 × 10^−1^	8.38 × 10^−3^	1.90 × 10^−1^
12-Hydroxyjasmonic acid	−6.70 × 10^−1^	1.28 × 10^−4^	9.03 × 10^−3^	−1.37 × 10^−1^	3.82 × 10^−1^	8.35 × 10^−1^	5.33 × 10^−1^	9.22 × 10^−3^	1.90 × 10^−1^
4-Nitroanthranilate	7.43 × 10^−1^	1.54 × 10^−4^	1.02 × 10^−2^	3.70 × 10^−1^	3.60 × 10^−2^	5.87 × 10^−1^	−3.73 × 10^−1^	1.03 × 10^−1^	4.17 × 10^−1^
2-Acetamido-3-(4-methoxyphenyl)propanoic acid	7.15 × 10^−1^	1.86 × 10^−4^	1.11 × 10^−2^	1.30 × 10^−1^	4.49 × 10^−1^	8.66 × 10^−1^	−5.86 × 10^−1^	8.98 × 10^−3^	1.90 × 10^−1^
Phenylethyl-β-D-galactopyranoside	−6.86 × 10^−1^	2.17 × 10^−4^	1.24 × 10^−2^	−3.16 × 10^−1^	5.76 × 10^−2^	6.82 × 10^−1^	3.69 × 10^−1^	8.79 × 10^−2^	3.95 × 10^−1^
Pyrrole-2-carboxylic acid	−7.00 × 10^−1^	2.35 × 10^−4^	1.25 × 10^−2^	−1.12 × 10^−1^	5.13 × 10^−1^	8.87 × 10^−1^	5.88 × 10^−1^	8.28 × 10^−3^	1.90 × 10^−1^
Adenosine 3′,5′-cyclic monophosphate	6.14 × 10^−1^	2.43 × 10^−4^	1.25 × 10^−2^	5.25 × 10^−1^	3.47 × 10^−4^	5.93 × 10^−2^	−3.11 × 10^−1^	1.12 × 10^−1^	4.35 × 10^−1^
Maleic acid	6.81 × 10^−1^	2.50 × 10^−4^	1.25 × 10^−2^	6.06 × 10^−2^	7.16 × 10^−1^	9.51 × 10^−1^	−6.21 × 10^−1^	4.42 × 10^−3^	1.72 × 10^−1^
Ala-Glu	6.76 × 10^−1^	2.63 × 10^−4^	1.25 × 10^−2^	2.36 × 10^−1^	1.56 × 10^−1^	7.86 × 10^−1^	−4.40 × 10^−1^	4.23 × 10^−2^	3.27 × 10^−1^
N-[(2S)-2-Hydroxypropanoyl]-L-phenylalanine	6.88 × 10^−1^	3.65 × 10^−4^	1.56 × 10^−2^	2.23 × 10^−1^	1.97 × 10^−1^	7.86 × 10^−1^	−4.65 × 10^−1^	3.96 × 10^−2^	3.21 × 10^−1^
3-(4-Hydroxy-3-methoxyphenyl)-2-oxiranecarboxylic acid	6.83 × 10^−1^	4.27 × 10^−4^	1.77 × 10^−2^	2.87 × 10^−1^	9.91 × 10^−2^	7.64 × 10^−1^	−3.96 × 10^−1^	8.08 × 10^−2^	3.91 × 10^−1^
L,L-Diaminopimelate	−6.24 × 10^−1^	6.99 × 10^−4^	2.54 × 10^−2^	−1.02 × 10^−1^	5.36 × 10^−1^	8.91 × 10^−1^	5.21 × 10^−1^	1.56 × 10^−2^	2.36 × 10^−1^
Methyl 2-(acetylamino)-4-amino-4-oxobutanoate	6.49 × 10^−1^	7.25 × 10^−4^	2.56 × 10^−2^	1.87 × 10^−1^	2.78 × 10^−1^	7.98 × 10^−1^	−4.62 × 10^−1^	3.98 × 10^−2^	3.21 × 10^−1^
3-Methyl-2,5-piperazinedione	−6.12 × 10^−1^	8.84 × 10^−4^	3.00 × 10^−2^	4.49 × 10^−2^	7.86 × 10^−1^	9.59 × 10^−1^	6.57 × 10^−1^	2.39 × 10^−3^	1.60 × 10^−1^
4-Methylene-L-glutamine	−6.12 × 10^−1^	9.02 × 10^−4^	3.00 × 10^−2^	−2.60 × 10^−1^	1.17 × 10^−1^	7.64 × 10^−1^	3.52 × 10^−1^	1.03 × 10^−1^	4.17 × 10^−1^
Fumaric acid	6.41 × 10^−1^	1.03 × 10^−3^	3.34 × 10^−2^	1.67 × 10^−1^	3.37 × 10^−1^	8.21 × 10^−1^	−6.97 × 10^−1^	2.41 × 10^−3^	1.60 × 10^−1^
4H-Pyran-4-one	6.36 × 10^−1^	1.18 × 10^−3^	3.73 × 10^−2^	−2.35 × 10^−1^	1.82 × 10^−1^	7.86 × 10^−1^	−8.71 × 10^−1^	1.67 × 10^−4^	4.21 × 10^−2^
3-Oxoglutaric acid	−6.04 × 10^−1^	1.22 × 10^−3^	3.75 × 10^−2^	−7.59 × 10^−1^	8.28 × 10−^6^	4.96 × 10^−3^	−2.56 × 10^−1^	2.07 × 10^−1^	5.59 × 10^−1^
6-Methylindole	−6.11 × 10^−1^	1.31 × 10^−3^	3.92 × 10^−2^	2.24 × 10^−1^	1.96 × 10^−1^	7.86 × 10^−1^	5.67 × 10^−1^	1.10 × 10^−2^	2.13 × 10^−1^
L-2-Aminooctanedioate	6.08 × 10^−1^	1.50 × 10^−3^	4.28 × 10^−2^	1.34 × 10^−1^	4.38 × 10^−1^	8.61 × 10^−1^	−4.74 × 10^−1^	3.46 × 10^−2^	3.02 × 10^−1^
2-O-Acetyl-3-O-trans-coutarate	−6.08 × 10^−1^	1.55 × 10^−3^	4.28 × 10^−2^	1.10 × 10^−1^	5.22 × 10^−1^	8.91 × 10^−1^	7.18 × 10^−1^	1.48 × 10^−3^	1.60 × 10^−1^
(2S,3R,4E,6E)-2-Amino-4,6-tetradecadiene-1,3-diol	−6.02 × 10^−1^	1.57 × 10^−3^	4.28 × 10^−2^	1.43 × 10^−3^	9.93 × 10^−1^	9.97 × 10^−1^	6.03 × 10^−1^	6.98 × 10^−3^	1.90 × 10^−1^
4,5-DiHDPE	−5.33 × 10^−1^	1.66 × 10^−3^	4.42 × 10^−2^	−1.21 × 10^−1^	4.26 × 10^−1^	8.58 × 10^−1^	4.12 × 10^−1^	3.82 × 10^−2^	3.16 × 10^−1^
6-Dehydro-SCB2	−5.82 × 10^−1^	1.76 × 10^−3^	4.43 × 10^−2^	−4.72 × 10^−2^	7.77 × 10^−1^	9.59 × 10^−1^	5.35 × 10^−1^	1.43 × 10^−2^	2.30 × 10^−1^
L-Kynurenine	5.83 × 10^−1^	1.82 × 10^−3^	4.43 × 10^−2^	4.61 × 10^−1^	8.28 × 10^−3^	3.93 × 10^−1^	−1.45 × 10^−1^	5.06 × 10^−1^	8.03 × 10^−1^
Deoxycholic acid	−6.08 × 10^−1^	1.92 × 10^−3^	4.43 × 10^−2^	−2.32 × 10^−1^	1.89 × 10^−1^	7.86 × 10^−1^	3.76 × 10^−1^	1.01 × 10^−1^	4.14 × 10^−1^
(±)12(13)-DiHOME	−6.10 × 10^−1^	1.92 × 10^−3^	4.43 × 10^−2^	−2.69 × 10^−1^	1.11 × 10^−1^	7.64 × 10^−1^	4.78 × 10^−1^	3.84 × 10^−2^	3.16 × 10^−1^
Hostmaniane	−5.66 × 10^−1^	1.93 × 10^−3^	4.43 × 10^−2^	−1.37 × 10^−1^	4.03 × 10^−1^	8.40 × 10^−1^	4.29 × 10^−1^	4.50 × 10^−2^	3.35 × 10^−1^
5-Hydroxyindole-3-acetic acid	5.79 × 10^−1^	1.95 × 10^−3^	4.43 × 10^−2^	5.34 × 10^−1^	2.18 × 10^−3^	3.00 × 10^−1^	−1.58 × 10^−1^	4.90 × 10^−1^	7.90 × 10^−1^
Lactamide	5.76 × 10^−1^	1.96 × 10^−3^	4.43 × 10^−2^	9.71 × 10^−2^	5.61 × 10^−1^	9.00 × 10^−1^	−4.79 × 10^−1^	2.82 × 10^−2^	2.78 × 10^−1^
L-Leucine	−5.55 × 10^−1^	2.01 × 10^−3^	4.47 × 10^−2^	−1.42 × 10^−1^	3.79 × 10^−1^	8.32 × 10^−1^	4.13 × 10^−1^	5.00 × 10^−2^	3.51 × 10^−1^
Perillic acid	−5.72 × 10^−1^	2.13 × 10^−3^	4.63 × 10^−2^	−7.49 × 10^−2^	6.55 × 10^−1^	9.43 × 10^−1^	4.98 × 10^−1^	2.29 × 10^−2^	2.62 × 10^−1^
cis-Aconitic acid	−4.65 × 10^−1^	1.25 × 10^−2^	1.25 × 10^−1^	−8.07 × 10^−1^	2.17 × 10−^6^	2.60 × 10^−3^	−3.42 × 10^−1^	1.17 × 10^−1^	4.50 × 10^−1^
cyclo (L-Glutamyl-L-glutamyl)	−5.24 × 10^−1^	5.40 × 10^−3^	7.80 × 10^−2^	−7.19 × 10^−1^	2.84 × 10^−5^	9.98 × 10^−3^	−1.95 × 10^−1^	3.78 × 10^−1^	7.18 × 10^−1^
Isethionic acid	−1.79 × 10^−1^	2.73 × 10^−3^	6.46 × 10^−2^	5.65 × 10^−1^	1.57 × 10^−2^	3.14 × 10^−1^	7.45 × 10^−1^	1.29 × 10^−4^	4.21 × 10^−2^
γ-L-Glutamyl-L-methionine	−5.50 × 10^−1^	4.24 × 10^−3^	6.83 × 10^−2^	2.92 × 10^−1^	9.19 × 10^−2^	7.64 × 10^−1^	8.42 × 10^−1^	2.11 × 10^−4^	4.21 × 10^−2^

* NW × OO is the interaction effect reflecting, if significant, differential response to asthma between the two groups, statistically given as (OO_A vs. OO)—(NW_A vs. NW). Only features with an FDR ≤ 0.05 in either NW or OO or an interaction effect NW × OO with a nominal *p* value ≤ 0.05 are shown. Abbreviations: NW, Normal weight without asthma; NW_A, Normal weight with asthma; OO, Overweight/obesity without asthma; OO_A, Overweight/obesity with asthma.

**Table 4:** Lipidomics association with asthma in NW and OO with correction for confounders.

Lipid	NW_A vs. NW			OO_A vs. NW			NW × OO *		
	Effect Size	*p* Value	FDR	Effect Size	*p* Value	FDR	Effect Size	*p* Value	FDR
Cer (d18:0_22:0)	−7.15 × 10^−1^	5.17 × 10^−5^	1.38 × 10^−2^	−1.99 × 10^−1^	2.16 × 10^−1^	8.72 × 10^−1^	−5.17 × 10^−1^	1.32 × 10^−2^	3.34 × 10^−1^
PE (18:1e_22:6)	−7.20 × 10^−1^	9.36 × 10^−5^	2.00 × 10^−2^	1.81 × 10^−1^	2.90 × 10^−1^	8.79 × 10^−1^	−7.35 × 10^−1^	7.65 × 10^−4^	1.84 × 10^−1^
PE (20:0p_22:6)	−6.66 × 10^−1^	1.62 × 10^−4^	2.89 × 10^−2^	1.25 × 10^−2^	9.38 × 10^−1^	9.85 × 10^−1^	−6.78 × 10^−1^	1.19 × 10−^3^	1.84 × 10^−1^
PC (20:4_22:6)	−6.93 × 10^−1^	2.19 × 10^−4^	3.35 × 10^−2^	−3.32 × 10^−1^	5.89 × 10^−2^	7.16 × 10^−1^	−4.38 × 10^−1^	4.77 × 10^−2^	4.52 × 10^−1^
PC (18:2e_19:0)	−6.66 × 10^−1^	3.43 × 10^−4^	4.22 × 10^−2^	−1.95 × 10^−1^	2.48 × 10^−1^	8.72 × 10^−1^	−4.70 × 10^−1^	3.22 × 10^−2^	4.28 × 10^−1^
SM (d18:1_23:0)	−6.77 × 10^−1^	3.55 × 10^−4^	4.22 × 10^−2^	−2.05 × 10^−1^	2.34 × 10^−1^	8.72 × 10^−1^	−4.72 × 10^−1^	3.51 × 10^−2^	4.38 × 10^−1^
PE (18:2_18:2)	−6.00 × 10^−1^	6.40 × 10^−4^	6.35 × 10^−2^	2.26 × 10^−1^	1.58 × 10^−1^	8.44 × 10^−1^	−8.27 × 10^−1^	7.97 × 10^−5^	4.26 × 10^−2^

* NW × OO is the interaction effect reflecting, if significant, differential response to asthma between the two groups, statistically given as (OO_A vs. OO)—(NW_A vs. NW). Only features with an FDR ≤ 0.05 in either NW or OO or an interaction effect NW × OO with a nominal *p* value ≤ 0.05 are shown. Abbreviations: Cer, Ceramide; NW, Normal weight without asthma; NW_A, Normal weight with asthma; OO, Overweight/obesity without asthma; OO_A, Overweight/obesity with asthma; PE, Phosphatidyl ethanolamine; PC, Phosphatidyl choline; SM, Sphingomyelin.

**Table 5:** Asthma-associated effects identified in the SOAP pediatric cohort and replicated in the QBB adult cohort.

Lipid	SOAP		QBB		Meta-Analysis					
	Effect	*p* Value	Effect	*p* Value	Effect	*p* Value	FDR	CI_lb *	CI_ub *	Contrast
LPC (16:0)	−3.58 × 10^−1^	3.63 × 10^−2^	−3.23 × 10^−1^	7.09 × 10^−3^	−3.35 × 10^−1^	6.35 × 10^−4^	5.71 × 10^−3^	−5.27 × 10^−1^	−1.43 × 10^−1^	OO_A vs. OO
18:0 SM (d18:1/18:0)	−3.66 × 10^−1^	4.57 × 10^−2^	−5.01 × 10^−1^	1.40 × 10^−2^	−4.27 × 10^−1^	1.71 × 10^−3^	1.37 × 10^−2^	−6.93 × 10^−1^	−1.60 × 10^−1^	NW_A vs. NW
Palmitoylcarnitine	−3.93 × 10^−1^	2.65 × 10^−2^	−2.58 × 10^−1^	2.87 × 10^−2^	−2.99 × 10^−1^	2.22 × 10^−3^	1.55 × 10^−2^	−4.91 × 10^−1^	−1.07 × 10^−1^	OO_A vs. OO
LPC (20:4)	−4.20 × 10^−1^	1.44 × 10^−2^	−2.10 × 10^−1^	8.58 × 10^−2^	−2.81 × 10^−1^	4.65 × 10^−3^	2.79 × 10^−2^	−4.76 × 10^−1^	−8.64 × 10^−2^	OO_A vs. OO

* CI_lb/CI_ub are lower bound/upper bound confidence intervals for the effect size from the meta-analysis. Abbreviations: NW, Normal weight without asthma; NW_A, Normal weight with asthma; OO, Overweight/obesity without asthma; OO_A, Overweight/obesity with asthma.

## Data Availability

The metabolomics and lipidomics data and metadata of this study will be made available upon request to the corresponding author.

## Supplementary Materials

The following supporting information can be downloaded at: https://www.mdpi.com/article/10.3390/metabo16050333/s1, Supplementary Table S1: General characteristics of study participants; Supplementary Table S2: Phenotypes showing nominally significant association with asthma and obesity; Supplementary Table S3: Metabolites showing nominally significant association with asthma and obesity; Supplementary Table S4: Lipids showing nominally significant association with asthma and obesity; Supplementary Table S5: General characteristics of QBB study participants.

## Abbreviations

The following abbreviations are used in this manuscript:

BMI	Body mass index
FEF 25–75%	Forced expiratory flow at 25% and 75% of the pulmonary volume
FeNO	Fractional exhaled nitric oxide
FET	Forced expiratory time
FEV1	Forced expiratory volume in 1 s
FRC	Functional residual capacity
FVC	Forced vital capacity
ERV	Expiratory reserve volume
IC	Inspiratory capacity
LCI	Lung clearance index
LPC	Lysophophatidylcholine
PE	Phosphatidylethanolamines
PEF	Peak expiratory flow
PIF	Peak inspiratory flow
RV	Residual volume
SM	Sphingomyelins
sRAW	Specific airway resistance
TCA	Tricarboxylic acid
TLC	Total lung capacity
VC	Vital capacity
