# Supplementary material for: A Multi-Omics Approach Uncovers Divergent Mechanisms of Asthma in Normal Weight and Obese Children
Source: Metabolites. 2026 May 15;16(5):333. doi: 10.3390/metabo16050333 (PMC13208224; doi:10.3390/metabo16050333)
Supplement: Supplementary file 1 [file metabolites-16-00333-s001.zip › Supplementary table S5.pdf]

Supplementary Table S5. General characteristics of QBB study participants.

	**NW_A (*n* = 95)**	**NW (*n* = 67)**	**OO_A (*n* = 99)**	**OO (*n* = 100)**	***p* value** (NW_A x NW)	***p* value** (OO_A x OO)
Gender	M:38, F: 37	M:504, F:230	M:18, F:8	M:154, F:80	0.0028	0.82
Age (year)	41 (31-47)	39 (30-49)	40 (34-52)	39 (30-48)	0.54	0.028
BMI	28 (25-32)	28 (25-33)	27 (25-29)	28 (25-32)	0.188	0.86

Abbreviations: BMI, Body mass index; *NW_A*, Normal wight with asthma; *OO*, Overweight/obesity without asthma; *OO_A*, Overweight/obesity with asthma
