# Supplementary material for: A Multi-Omics Approach Uncovers Divergent Mechanisms of Asthma in Normal Weight and Obese Children
Source: Metabolites. 2026 May 15;16(5):333. doi: 10.3390/metabo16050333 (PMC13208224; doi:10.3390/metabo16050333)
Supplement: Supplementary file 1 [file metabolites-16-00333-s001.zip › Supplementary table S1.pdf]

Supplementary table S1. General characteristics of study participants

Variables	NW-A (*n* = 95)	NW (*n* = 67)	OO-A (*n* = 99)	OO (*n* = 100)	*p* value (NW_A_x_NW)	*p* value (OO_A_x_OO)
Gender (n)	M:70, F: 25	M: 36 F: 31	M: 72, F: 27	M: 45 F: 55	1.17E-02	9.29E-05
Age (year), mean (range)	10 (8-13.5)	10 (8-13)	12 (9-15)	13 (10.5-14.5)	6.24E-01	5.57E-01
Weight (kg), mean (range)	31.2 (25.9-46.2)	31.77 (24.57-44.05)	60.15 (44.5-81.8)	76.8 (61.08-95.07)	5.18E-01	<1E-04
BMI (kg/m^2), mean (range)	17 (15-19)	16 (15-18)	26 (22.5-30)	31 (25-38)	5.60E-01	<1E-04
BMI percentile, mean (range)	45 (21-69)	38.5 (17-58.75)	97 (94-99)	98.5 (97-100)	7.50E-03	<1E-04
Fat (%), mean (SD)	16.67 (5.7)	17.44 (5.86)	33.42 (8.59)	40.24 (10.72)	4.61E-01	<1E-04
Fat mass (kg), mean (range)	5 (4-8)	5 (4-7)	18 (13-30)	31 (18-43.5)	9.75E-01	<1E-04
FFM (kg), mean (range)	25.5 (21-38.75)	26 (20-35.5)	39 (28-52)	46 (36.5-52)	3.94E-0.1	1.53E-01
BMR (kcal/day), mean (range)	1158.5 (1067.25-1409.25)	1163.5 (1057.75-1333)	1530 (1301-1865)	1696 (1508.5-1940)	8.07E-0.1	7.3E-0.3
SBP (mmHg), mean (SD)	106.18 (9.65)	103.55 (8.64)	113.52 (8.92)	114.55 (10.31)	1.01E-01	5.23E-01
DBP (mmHg), mean (SD)	66.6 (6.92)	67.47 (7.61)	72.52 (6.29)	72.19 (7)	5.13E-01	7.60E-01

Data are presented as median (IQR) or mean (SD) after rank-normal transformation.

Abbreviations: BMI, Body mass index; *BMR*, basic metabolic rate; *FFM*, Free fat mass; *DBP*, Diastolic blood pressure; *NW_A*, Normal wight with asthma; *OO*, Overweight/obesity without asthma; *OO_A*, Overweight/obesity with asthma; *SBP;* systolic blood pressure
